# Feasibility of laparoscopic gastrectomy for elderly gastric cancer patients: meta-analysis of non-randomized controlled studies

**DOI:** 10.18632/oncotarget.16691

**Published:** 2017-03-29

**Authors:** Liang Zong, Aiwen Wu, Wenyue Wang, Jingyu Deng, Susumu Aikou, Hiroharu Yamashita, Masahiro Maeda, Masanobu Abe, Duonan Yu, Zhiwei Jiang, Yasuyuki Seto, Jiafu Ji

**Affiliations:** ^1^ Department of Gastrointestinal Surgery, Graduate School of Medicine, University of Tokyo, Tokyo, Japan; ^2^ Department of Gastrointestinal Surgery, Key Laboratory of Carcinogenesis and Translational Research (Ministry of Education), Peking University Cancer Hospital & Institute, Beijing, China; ^3^ Department of Gastrointestinal Surgery, Su Bei People’s Hospital of Jiangsu Province, Yangzhou University, Yangzhou, China; ^4^ Department of Gastrointestinal Surgery, China-Japan Friendship Hospital, Beijing, China; ^5^ Department of Gastroenterology, Tianjin Medical University Cancer Hospital, City Key Laboratory of Tianjin Cancer Center and National Clinical Research Center for Cancer, Tianjin, China; ^6^ Department of Gastrointestinal Surgery, Kyoto University Graduate School of Medicine, Kyoto, Japan; ^7^ Division for Health Service Promotion, University of Tokyo, Tokyo, Japan; ^8^ Institute of Comparative Medicine, Yangzhou University, Yangzhou, China; ^9^ Research Institute of General Surgery, Jinling Hospital, Nanjing, China

**Keywords:** gastric cancer, elderly patient, laparoscopic gastrectomy, open gastrectomy, meta-analysis

## Abstract

The aim of this meta-analysis was to determine the feasibility of laparoscopic gastrectomy (LG) for elderly gastric cancer patients by comparing laparoscopic and conventional open gastrectomies (OG). Comprehensive search of the PubMed, EMBASE, and Cochrane Library databases revealed nine non-randomized controlled studies that compared LG and OG in elderly gastric cancer patients We then analyzed dichotomous or continuous parameters using odds ratios (ORs) or weighted mean differences (WMDs). Overall survival was estimated using hazard ratios (HRs) with a fixed effects or random effects model. We observed that the age distribution was similar between the LG and OG patient groups (WMD -0.22 95% CI, -1.26−0.82). LG patients experienced less blood loss (WMD -119.14 95% CI, -204.17−-34.11) and had shorter hospital stays (WMD -3.48 95% CI, -5.41−-1.56), but endured longer operation times (WMD 10.87 95% CI, 2.50−19.24). Postoperatively, LG patients exhibited lower incidences of postoperative morbidities (OR 0.59 95% CI, 0.43−0.79), surgery related morbidities (OR 0.58 95% CI, 0.41−0.81) and systemic morbidities (OR 0.56 95% CI, 0.38−0.82). We observed no differences between the LG and OG patient groups regarding anastomotic leakage (OR 0.69 95% CI, 0.34−1.41), mental disease (OR 0.72 95% CI, 0.37−1.41) and long term effects (HR 0.98 95% CI, 0.74−1.32). We therefore conclude that laparoscopic gastrectomy might be technically feasible for elderly gastric cancer patients.

## INTRODUCTION

A recent multicenter randomized and controlled trial by Hu *et al* demonstrated that laparoscopic gastrectomy (LG) with D2 lymph node dissection was more feasible than the conventional open distal gastrectomy for advanced gastric cancer patients [1]. In our previous meta-analysis, we demonstrated that minimal invasive surgery, laparoscopic and robotic surgeries were all technically more feasible than open resections for gastric cancer because of their affirmative role in both subtotal and total gastrectomies [2]. Therefore, the feasibility of LG for elderly patients needs to be established since their numbers are rapidly increasing [3–5].

Generally, elderly patients are at a high risk for major surgeries because functional reserves decrease with age. Besides, systemic stress and inflammatory responses contribute to a higher risk of delirium for elderly patients, especially after surgery. Therefore elderly patients can benefit by undergoing LG because it is associated with less trauma, early bowel movement, and shorter hospital stay. However, issues like prolonged operation time and the impact of carbon dioxide pneumoperitoneum on circulatory and respiratory dynamics during LG procedure have not been evaluated [6–8].

Recently, many studies have investigated the application of LG for elderly gastric cancer patients. However, some retrospective studies have ignored selection bias while comparing outcomes in older and younger patients [9–22]. Limited sample sizes, single institution design and different appraise system of complications have impeded definitive conclusions in previous studies that have compared laparoscopic and open gastrectomy for elderly gastric cancer patients [23–31]. A recent meta-analysis by Wang *et al* evaluated the short term effects of LG for elderly gastric cancer patients by summarizing the studies that compared laparoscopic versus open gastrectomy for elderly gastric cancer patients [32]. However, the incidence of delirium which is a major concern in elderly patients undergoing surgery was not addressed. Also, their inclusion criteria were not stringent (e.g. no language limitation for included studies), which may have increased the heterogeneity of results. Moreover, they did not compare the long-term effects of LG vs. OG for elderly gastric cancer patients. Therefore, in this study, we performed an updated pooled analysis of studies that compared laparoscopic versus open gastrectomy for elderly gastric cancer patients.

## RESULTS

### Study eligibility

**Figure 1:**
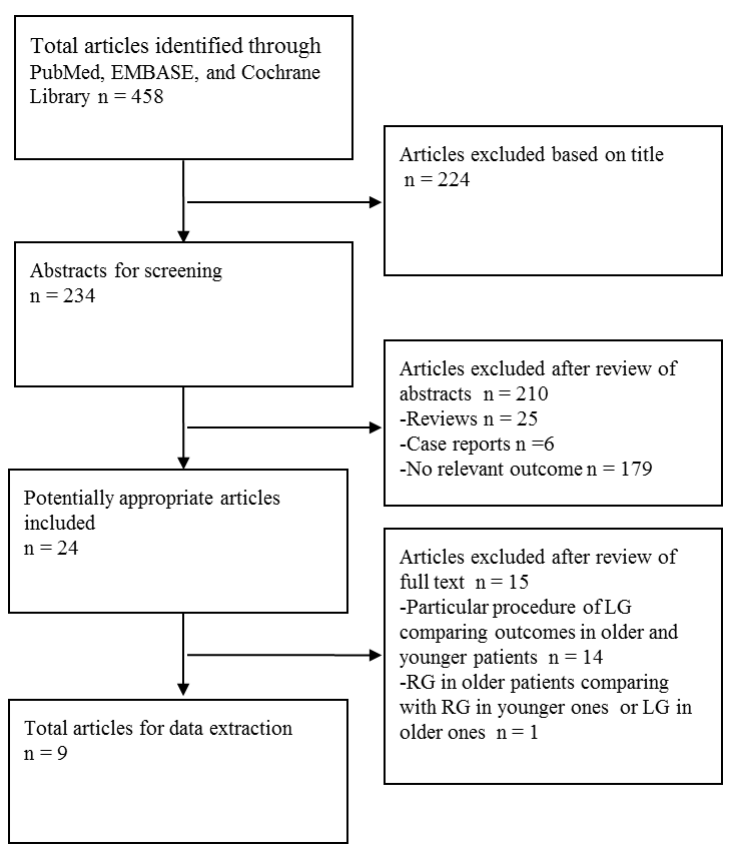
Flow chart of literature selection

Initially, we identified 458 articles upon literature survey in three databases, PubMed, EMBASE and Cochrane library (Figure 1). Next, 434 articles were excluded after title and abstract screening using the exclusion criteria. Further, 14 articles comparing outcomes in older and younger patients regarding a particular procedure of LG were also excluded (Supplementary Table 1) [9–22]. Another article that compared robotic gastrectomy for elderly gastric cancer patients with robotic gastrectomy in younger patients or laparoscopic gastrectomy in the elderly was also excluded [33]. Finally, nine studies were selected for further meta-analysis [23–31]. Two of the nine studies compared data of a particular procedure of LG in older and younger patients (Supplementary Table 1) [30–31].

### Study characteristics

The nine studies for the meta-analysis included three prospective and six retrospective studies [23–31]. Supplementary Table 2 lists the main characteristics of the nine studies. The sample size in the studies ranged from 46 to 504 (Figure 1). Of the nine studies, 7 analyzed age distribution, 6 analyzed operation time or blood loss, 5 analyzed hospital stay, 4 analyzed lymph node harvest or overall survival, 8 analyzed postoperative complications, 7 analyzed surgery related morbidity or anastomotic leakage or systemic morbidities and 3 analyzed mental disease.

**Figure 2:**
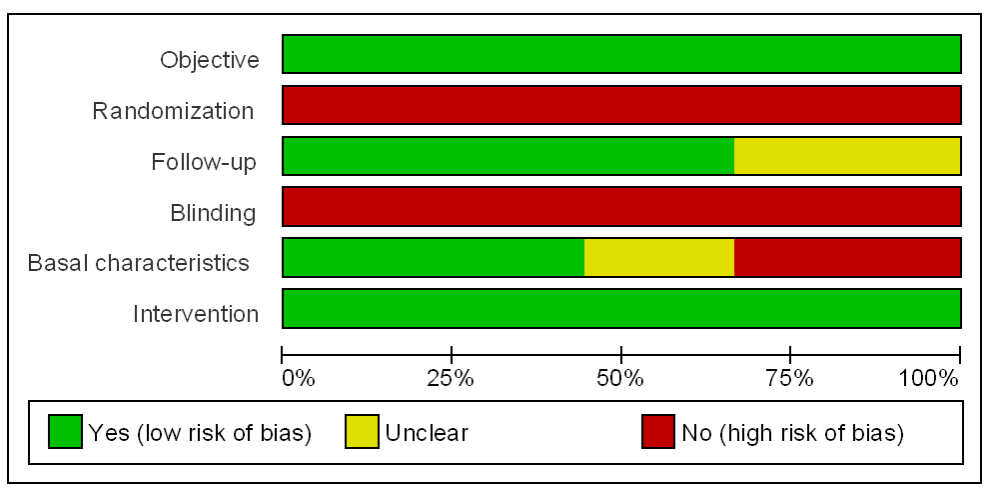
Analysis of risk of bias of each included study Green color denotes studies with low risk of bias; red color denotes studies with high risk of bias; yellow color denotes studies with insufficient information for assessing risk of bias.

Figure 2 lists the risk of bias of each included study from objective, randomization, follow-up, blinding, baseline characteristics, and intervention [34]. These six items were explained as follows: 1) Objective: Does the study clearly answer the defined question? 2) Randomization: Were the elderly patients randomly assigned to open or laparoscopic group? 3) Follow-up: Was the number of patients enrolled at the start of the study recorded? Was the end point of the study clearly indicated? 4) Blinding: Were the researchers and the elderly patients blinded with respect to the surgical method? 5) Baseline characteristics: Were the studied elderly patients similar at the start of the study? 6) Intervention: Were the groups treated in the same way except for experimental intervention?

**Figure 3:**
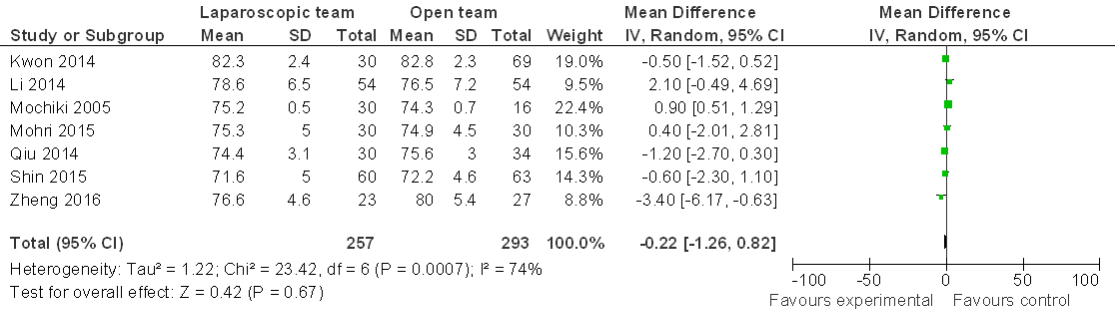
Comparison of age distribution between LG and OG Random effect meta-analysis shows similar age distribution between the two groups.

Eight of the nine included studies defined an age over 65 or 70 as elderly. One study by Kwon *et al* defined age over 80 as elderly [35]. Our analysis showed that age distribution between LG and OG procedures was similar (WMD -0.22 95% CI, -1.26−0.82; *P* = 0.67; Figure 3).

### Comparisons of general parameters between LG and OG

**Figure 4:**
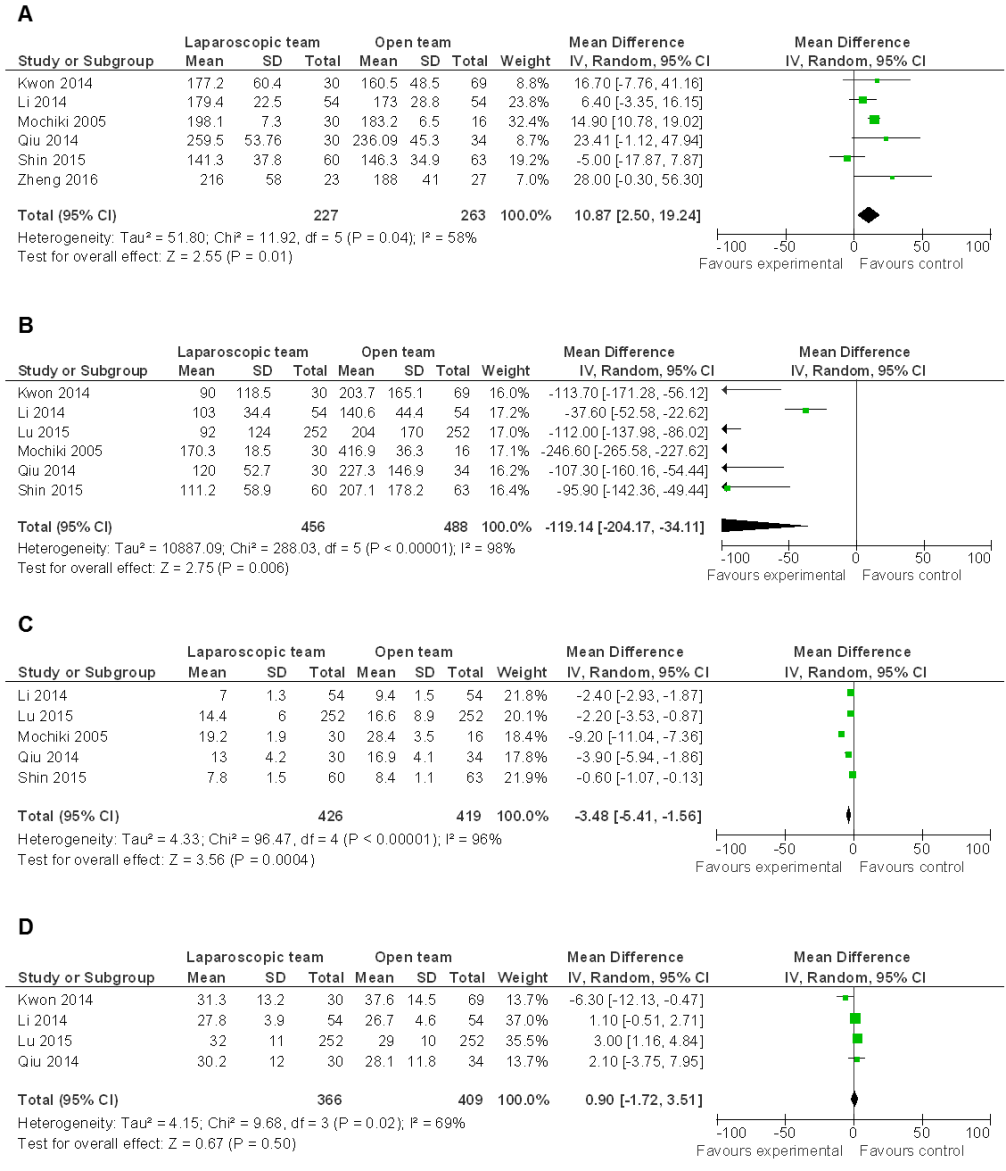
Comparison of general parameters related to LG and OG **A.-C**. Random effect meta-analysis shows more operation time low blood loss and less hospital stay in LG. **D.** Random effect meta-analysis shows no difference in lymph node harvest between the two groups.

The mean operation time of LG was longer than OG (WMD 10.87 95% CI, 2.50−19.24; *P* = 0.01) (Figure 4A). However, intraoperative blood loss (WMD -119.14 95% CI, -204.17−-34.11; *P* = 0.006) (Figure 4B) and hospital stay (WMD -3.48 95% CI, -5.41−-1.56; *P* = 0.0004) (Figure 4C) were significantly reduced by LG. The differences in lymph node harvest between LG and OG were not statistically significant (WMD 0.90, 95% CI, -1.72−3.51; *P* = 0.50) (Figure 4D).

### Comparisons of postoperative complications between LG and OG

**Figure 5:**
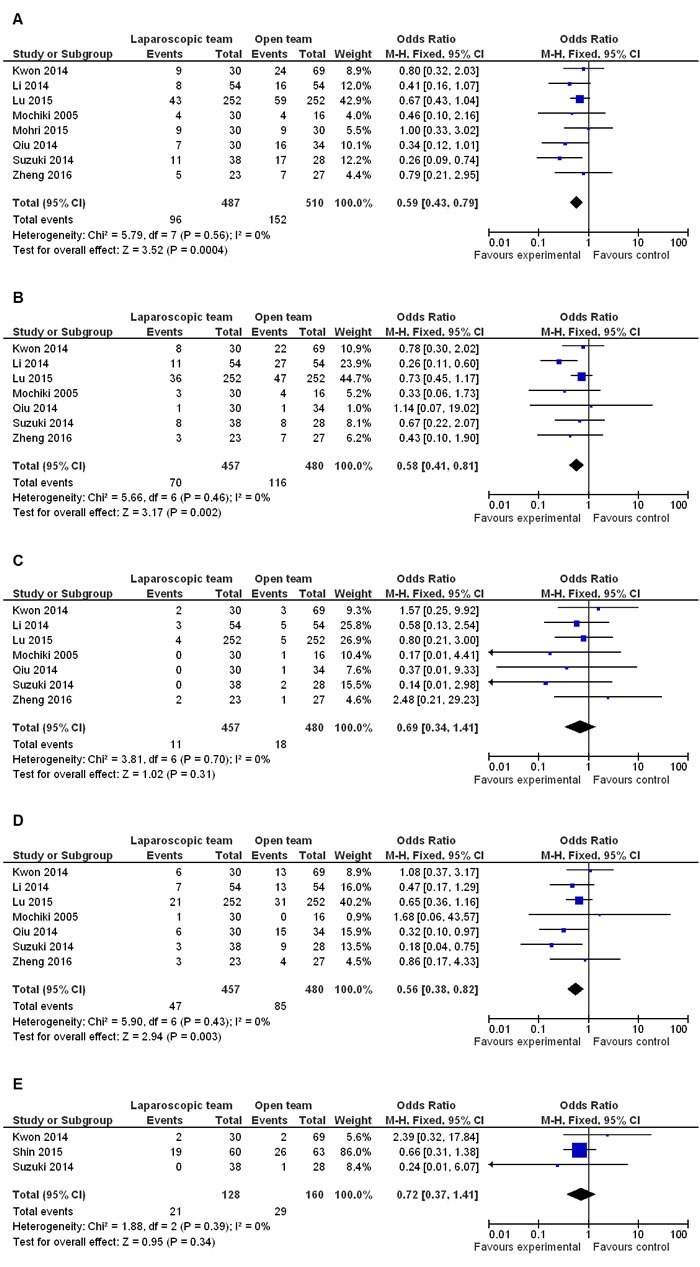
Comparison of postoperative complications in LG and OG Fixed effect meta-analysis shows that **A.** LG significantly reduces the incidence of postoperative complications compared to OG; **B.-C**. Surgery related morbidity is lower in LG, whereas anastomotic leakage is similar between LG and OG groups; and **D.-E**. LG reduces the incidence of systemic morbidities, whereas no difference is observed regarding mental disease between the two groups.

Meta-analysis on total postoperative complications showed that LG significantly reduced their incidence compared to OG (OR 0.59 95% CI, 0.43−0.79; *P* = 0.0004) (Figure 5A). Moreover, total surgery related morbidities like wound infection, intra-abdominal abscess, bleeding, leakage, stricture and bowel obstruction were reduced in LG compared to OG (OR 0.58 95% CI, 0.41−0.81; *P* = 0.002) (Figure 5B). However, anastomotic leakage was similar between the two procedures (OR 0.69 95% CI, 0.34−1.41; *P* = 0.31) (Figure 5C). Further, meta-analysis data revealed that LG reduced the incidence of total systemic morbidities like respiratory, cardiological, enterocolitis, urinary, and mental disease (OR 0.56 95% CI, 0.38−0.82; *P* P = 0.003) (Figure 5D). However, no differences were observed between the two groups regarding mental disease (OR 0.72 95% CI, 0.37−1.41; *P* = 0.34) (Figure 5E).

### Analysis of overall survival in LG and OG patient groups

**Figure 6:**
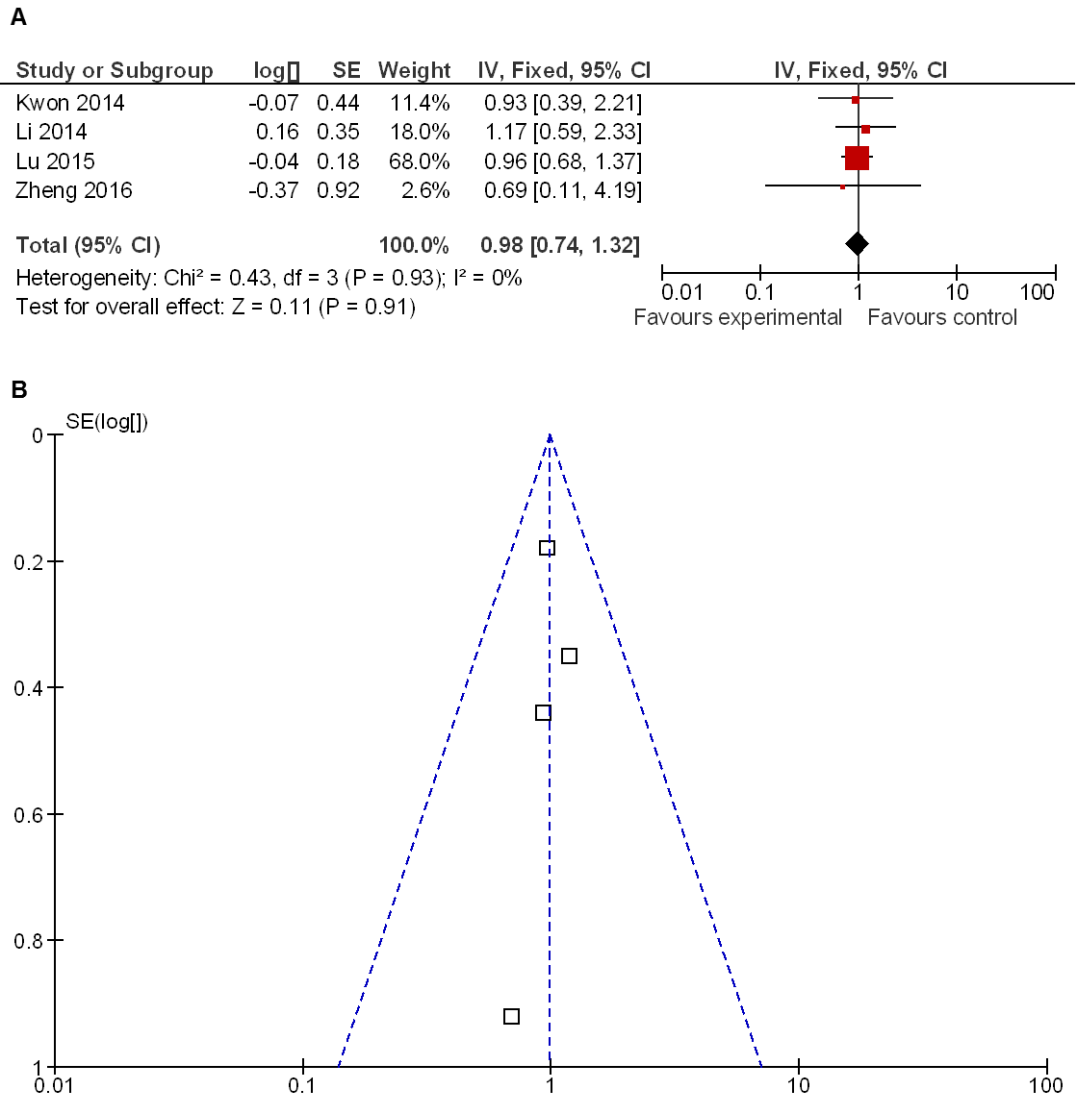
Overall survival rates in LG and OG groups of patients **A.** Meta-analysis shows that overall survival rates are similar for both LG and OG groups. **B.** Funnel plot shows that no heterogeneity exists among four included studies.

Meta-analysis showed that overall survival of LG and OG patients was similar (HR 0.98 95% CI, 0.74−1.32; *P* = 0.91) (Figure 6A). Funnel plot showed no heterogeneity among the four included studies that reported overall survival (Figure 6B).

## DISCUSSION

In this study, we reviewed the data from nine studies to estimate the feasibility of LG for the elderly gastric cancer patients. We observed that although LG patients endured more operation time there was less intraoperative bleeding and shorter hospital stay in LG compared to OG. Further, lymph node harvest times were similar between LG and OG. Interestingly, LG significantly reduced the incidence of postoperative complications. Although anastomotic leakage was similar between two groups, our analysis demonstrated that incidences of surgery related and systemic morbidities were greatly reduced in LG. Moreover, no differences existed between the two groups in regard to mental disease. Overall survival between LG and OG patients was comparable, thereby suggesting LG is technically feasible for the elderly patients.

A major confounding factor in the systematic review on topics relating to elderly patients was the lack of a standardized definition. The definition of ‘elderly’ varies depending on the country. Supplementary Tables 1 and 2 list the characteristics of included and excluded studies, respectively. In Asian countries, age over 65 or 70 was considered as elderly, whereas in Western countries age over 60 was considered elderly. However, eight out of nine studies included for pooled analysis were conducted in Japan, Korea and China and were relatively consistent regarding age definition. The exception was a study by Kwon *et al* that defined the elderly as over 80 years old [23].

Another issue was the selection bias regarding the type of surgery between the open and laparoscopic surgeries. Depending on the preference of surgeons, the total or distal gastrectomy was more in demand in the laparoscopic surgery. We performed a meta-analysis of total or distal gastrectomy between laparoscopic and open surgeries to test if there was a selection bias of surgery type among the included studies. However, our analysis demonstrated no statistical difference between the two groups (Supplementary Figures 5 and 6).

Owing to shorter hospital stays and lower postoperative complication rates, laparoscopic surgery is widely performed for gastric cancer patients, especially in early stage patients [35]. Several studies have indicated that LG is safe and feasible, although it has not yet been established. In a large-scale (*n* = 1256), phase III, multicenter, prospective randomized controlled trial (KLASS-01) for stage I gastric cancer in Korea, it was observed that the overall complications in patients that underwent the laparoscopic distal gastrectomy was significantly lower than in patients that underwent open distal gastrectomy (13.0% vs. 19.9%, *P* = 0.001) [36]. This suggested that LG was safe for elderly patients with early stage gastric cancer [36]. Moreover, a matched cohort study showed no difference in 3-year survival rates in the advanced gastric cancer patients that underwent either laparoscopic or open surgery [37]. A recent multi-center RCT reported that laparoscopic procedure with D2 lymph node dissections was safer compared to conventional open distal gastrectomy for advanced gastric cancer [1]. In our study, both OG and LG achieved similar number of lymph node harvest.

Since elderly patients have decreased functional reserve and an increased number of comorbidities, there has been uncertainity regarding the application of LG until now. The main concern in the elderly regarding the use of LG is because of the possibility of cardiopulmonary dysfunction related to pneumoperitoneum. Preoperative comorbidities are also observed frequently in elderly patients undergoing conventional open gastrectomy. In a pooled analysis, preoperative comorbidities in LG were similar to those in OG (Supplementary Figure 1). However, postoperative data suggested that successful laparoscopic surgeries for elderly patients have reduced incidence of pneumoperitoneum-related respiratory and cardiovascular disease compared to OG (Supplementary Figures 2 and 3). Also, patients that underwent LG demonstrated reduced postoperative systemic morbidities.

Postoperative pain is mostly associated with postoperative delirium because surgical trauma induces inflammatory cytokines and cortisol in the nervous system that can alter cognitive function. It was observed that laparoscopic surgery was more advantageous than open surgery because it suppresses the intra-operative inflammatory responses [38]. Patients that underwent laparoscopic gastrectomy had significantly lower IL-6 and C-reactive protein [38]. LG also minimized damage from surgical trauma (Supplementary Figure 4). Hence, since minimal incision was involved in LG, we postulated favourable cognitive outcomes compared to OG. However, we observed no significant differences in the incidence of postoperative delirium between the two groups. This could be because of a complex interplay between predisposing (patient vulnerability) and precipitating (anesthetic, operative, and postoperative) factors that determine postoperative delirium.

Although we used stringent inclusion criteria, many confounding factors such as source of patients, varying definition of the elderly, surgical level of operator and publication bias limited us from making more precise conclusions. Lack of randomized controlled studies was a significant weakness of our study. Moreover, all the included studies in our meta-analysis were from East Asia that can be a source of selection bias. Also, significant heterogeneity among the data from different studies may have influenced the overall effect sizes even though the randomized model was used. To overcome these limitations, a multi-center randomized controlled trial with greatly increased sample sizes is needed in future.

In summary, this meta-analysis highlights that LG significantly reduces both surgery related and systemic morbidities compared with OG and does not increase cardiopulmonary or mental dysfunctions. Thus, our study demonstrates that laparoscopic gastrectomy might be technically feasible and advantageous for elderly patients.

## MATERIALS AND METHODS

### Publication search and inclusion criteria

We followed the standard guidelines to conduct this meta-analysis. First, we searched three databases (PubMed, EMBASE and Cochrane library) for studies that addressed the feasibility of laparoscopic gastrectomy for elderly gastric cancer patients until July 01, 2016. We used the following MeSH terms: *elderly*, *open*, *laparoscope or laparoscopic*, *gastrectomy*, and *cancer* or *carcinoma* or *adenocarcinoma*. All eligible studies in English were retrieved and their bibliographies were checked for relevant publications by two junior reviewers (Z. L. and D. J.). Further, both the reviewers screened the abstracts using exclusion criteria to exclude irrelevant articles. Then, the two reviewers evaluated the remaining full articles to ensure they satisfied all inclusion criteria.

Studies were excluded if (1) they were case reports, reviews, letters or editorials and lacked control groups; (2) they compared laparoscopic vs. open gastrectomy for elderly patients with benign lesions; (3) they reported a procedure comparing outcomes in older and younger patients.

Studies were included if (1) they evaluated efficacy of laparoscopic gastrectomy in elderly patients; (2) they were a randomized controlled trial (RCT) or non-RCT of LG *vs*. OG for elderly gastric cancer patients; (3) elderly were defined as over 60 years old; (4) only the latest or completed study was included if there was an overlap of authors or centers; and (5) there was clear published data of focused parameters to estimate an odds ratio (OR) with 95% confidence interval (CI).

### Quality assessment of included studies

Since there were no randomized controlled trials (RCT) included in this meta-analysis, methodological index for non-randomized studies (MINORS) was used to evaluate the quality of included studies [39]. This tool includes 12 items to evaluate the quality of non-randomized study.

### Data extraction

Two investigators (L.Z. and J.D.) independently assessed publications for inclusion in the study based on the criteria described previously. Discrepancies between the two reviewers were resolved by discussion with the three senior authors (Z.J., Y.S. and J.J.). Data including baseline characteristics such as first author's surname, publication period, region, study type, gender, age distribution, reconstruction methods, preoperative commodities, TNM stage, and total number of patients in OG or LG group, respectively were extracted from the eligible studies.

### Statistical analysis

Odd ratios (OR) with 95% CI was used to analyze the dichotomous variables (e.g., post-operative morbidities) among surgical methods. Weighted mean difference (WMD) with 95% confidence intervals (95% CI) was used for continuous parameters like operation time and blood loss. The association between surgical methods and overall survival was evaluated using the weighted average of individually adjusted log hazard ratios (HRs), wherein the weights were inversely proportional to the variance of the log HR of each study. Statistical tests of P value and heterogeneity were analyzed as previously described [40]. All statistical tests were performed with Review Manager Version 5.0 software (The Cochrane Collaboration, Oxford, England).

## SUPPLEMENTARY MATERIALS FIGURES AND TABLES






