# Supplementary material for: Feasibility of laparoscopic gastrectomy for elderly gastric cancer patients: meta-analysis of non-randomized controlled studies
Source: Oncotarget. 2017 Mar 29;8(31):51878–87. doi: 10.18632/oncotarget.16691 (PMC5584298; doi:10.18632/oncotarget.16691)
Supplement: Supplementary file 1 [file oncotarget-08-51878-s001.pdf]

Feasibility of laparoscopic gastrectomy for elderly gastric cancer patients: meta-analysis of non-randomized controlled studies

Supplementary Material

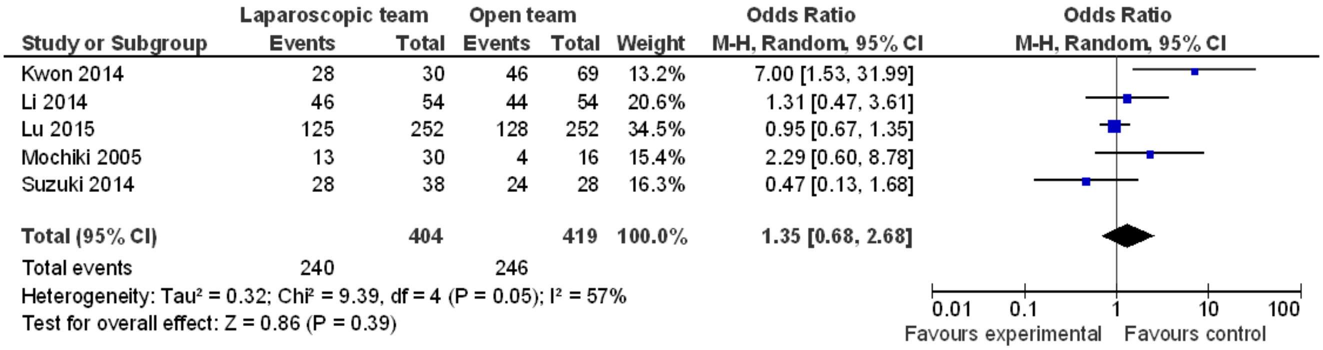

Supplementary Figure 1: Comparison of preoperative comorbidities between LG and OG. Random effect meta-analysis shows similar preoperative comorbidities between the two groups.

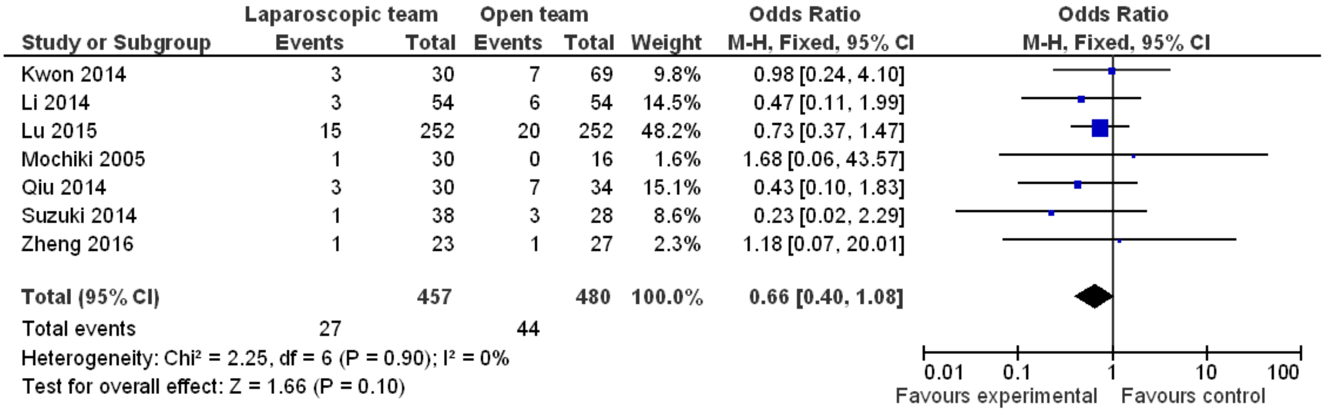

Supplementary Figure 2: Comparison of the postoperative respiratory diseases in LG and OG. Fixed effect meta-analysis shows no significant difference in postoperative respiratory diseases between the two groups.

Study or Subgroup	Laparoscopic team Events	Laparoscopic team Total	Open team Events	Open team Total	Weight	Odds Ratio M-H, Fixed, 95% CI
Kwon 2014	1	30	1	69	4.0%	2.34 [0.14, 38.78]
Li 2014	1	54	1	54	6.7%	1.00 [0.06, 16.41]
Lu 2015	2	252	3	252	20.5%	0.66 [0.11, 4.01]
Mochiki 2005	0	30	0	16		Not estimable
Qiu 2014	0	30	3	34	22.2%	0.15 [0.01, 2.98]
Suzuki 2014	3	38	4	28	29.2%	0.51 [0.11, 2.51]
Zheng 2016	2	23	3	27	17.3%	0.76 [0.12, 5.01]
**Total (95% CI)**		**457**		**480**	**100.0%**	**0.61 [0.27, 1.41]**
Total events	9		15			

Heterogeneity: Chi² = 1.97, df = 5 (P = 0.85); I² = 0%

Test for overall effect: Z = 1.15 (P = 0.25)

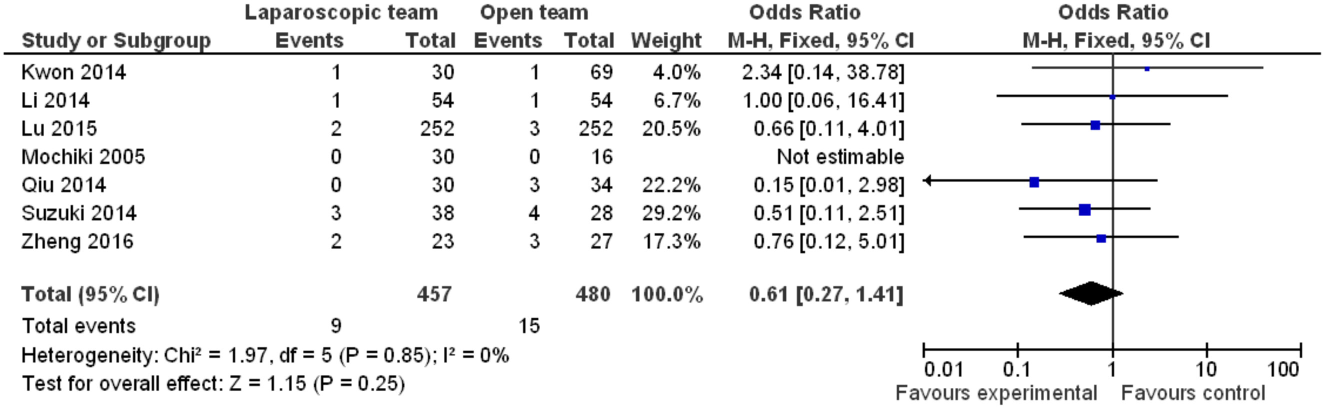

Supplementary Figure 3: Comparison of postoperative cardiovascular diseases in LG and OG patients. Fixed effect meta-analysis shows comparable postoperative cardiovascular diseases in LG.

Study or Subgroup	Laparoscopic team Events	Laparoscopic team Total	Open team Events	Open team Total	Weight	Odds Ratio M-H, Fixed, 95% CI
Kwon 2014	5	30	8	69	17.2%	1.52 [0.45, 5.12]
Li 2014	2	54	9	54	36.8%	0.19 [0.04, 0.94]
Lu 2015	2	252	6	252	25.3%	0.33 [0.07, 1.64]
Mochiki 2005	1	30	2	16	10.7%	0.24 [0.02, 2.89]
Qiu 2014	1	30	0	34	1.9%	3.51 [0.14, 89.42]
Suzuki 2014	1	38	0	28	2.3%	2.28 [0.09, 58.07]
Zheng 2016	0	23	1	27	5.8%	0.38 [0.01, 9.68]
**Total (95% CI)**		**457**		**480**	**100.0%**	**0.58 [0.30, 1.14]**
Total events	12		26			

Heterogeneity: Chi² = 7.22, df = 6 (P = 0.30); I² = 17%

Test for overall effect: Z = 1.58 (P = 0.11)

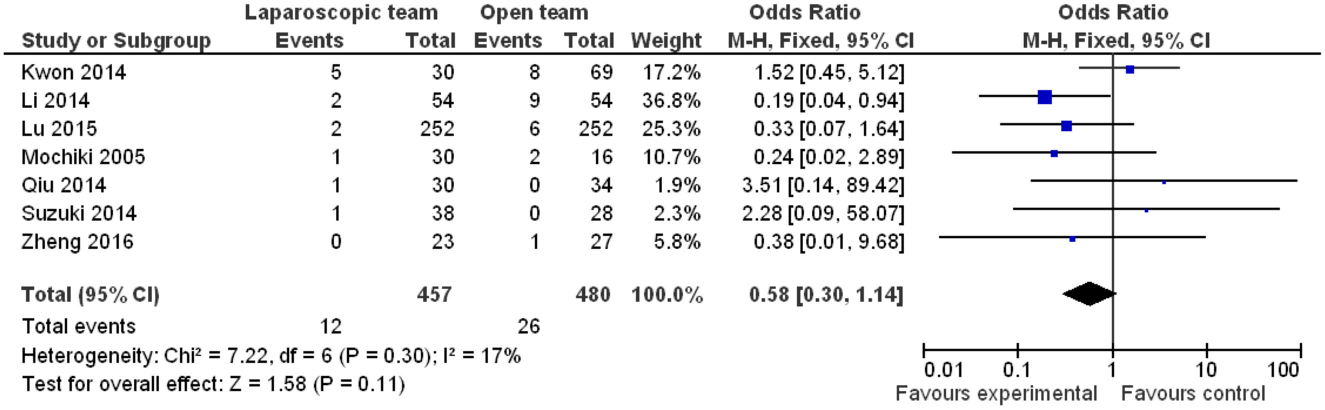

Supplementary Figure 4: Comparison of wound infections in LG and OG patients. Fixed effect meta-analysis shows similar incidence of wound infection between the two groups.

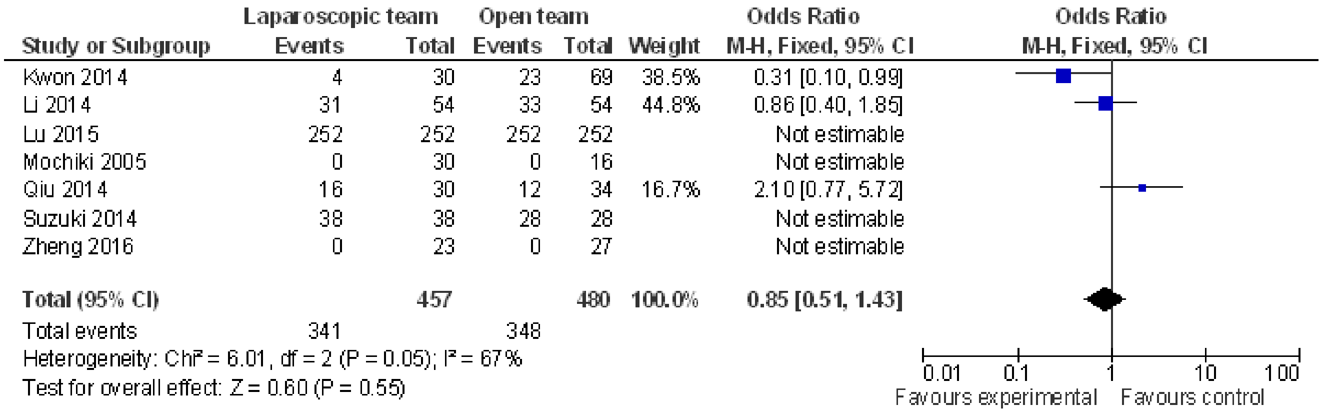

Supplementary Figure 5: Comparsion of the total number of gastrectomy in LG and OG patients. Fixed effect meta-analysis shows no differences between the two groups regarding total gastrectomy.

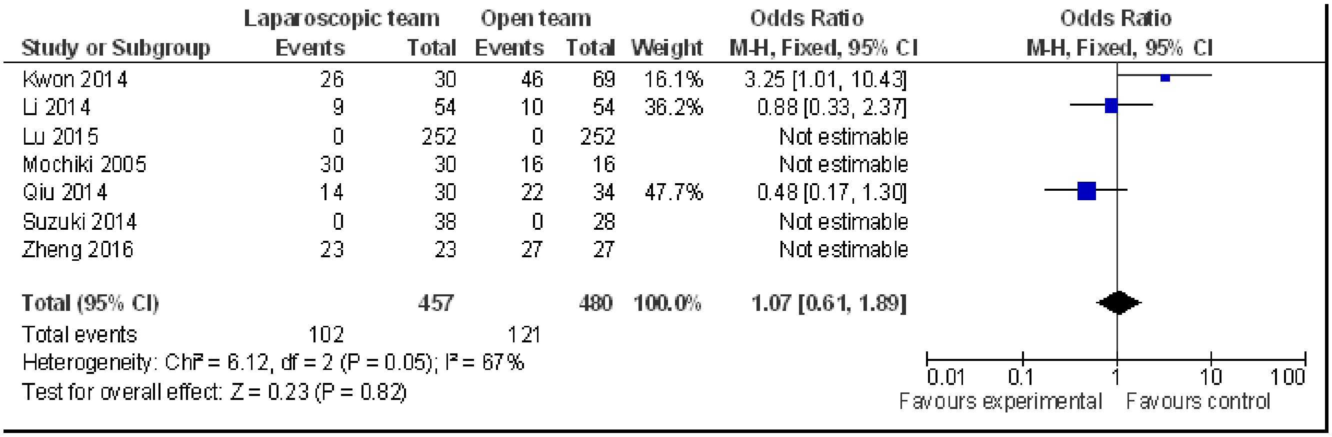

Supplementary Figure 6: Comparison of the total numbers of distal gastrectomy in LG and OG patients. Fixed effect meta-analysis shows no differences between the two groups regarding distal gastrectomy

For Supplementary Tables 1, 2 see in Supplementary Files
